# Case report: Co-occurrence of Wilson’s and Alexander’s diseases revealed by genetic analysis

**DOI:** 10.3389/fneur.2025.1514044

**Published:** 2025-03-12

**Authors:** Shufan Ge, Lanting Sun, Han Wang, Wenming Yang, Qiaoyu Xuan, Daiping Hua

**Affiliations:** ^1^Department of Neurology, The First Affiliated Hospital of Anhui University of Chinese Medicine, Anhui University of Chinese Medicine, Hefei, China; ^2^Center for Xin’an Medicine and Modernization of Traditional Chinese Medicine, Institute of Health and Medicine, Hefei Comprehensive National Science Center, Hefei, China

**Keywords:** Wilson disease, Alexander disease, concurrence, diagnosis, white matter lesions

## Abstract

Wilson’s disease (WD) and Alexander’s disease (AxD) are two prevalent genetic illnesses in clinical practice. However, cases of concurrent WD and AxD have not been reported. A mutation in the ATP7B gene causes improper copper metabolism, whereas AxD is caused by a mutation in the GFAP gene, which causes glial fibrillary acidic protein to accumulate in astrocytes. We present the first instance of concurrent WD and AxD in order to increase the diagnosis accuracy of this type of disease and provide a more precise treatment plan for the patient. A 10-year-old girl who appeared with diminished speech, limb weakness, trouble walking, and mental behavioral problems within the last 2 months. The patient’s copper biochemistry results and clinical manifestations supported the diagnosis of WD, however her uncommon bilateral frontal lobe cerebral white matter with considerable high signal in MRI differed from the normal neuroimaging presentations of WD. To clarify the patient’s diagnosis, we did whole-exome sequencing testing. To further clarify the patient’s diagnosis, we performed whole exome sequencing tests on the patient and her father and detected a single heterozygous mutation in the GFAP gene and a double heterozygous mutation in the ATP7B gene, with the two variant loci located on the same allele. Combined with the Leipzig score and characteristic MRI changes, the patient was diagnosed with co-morbid WD and AxD. The overlapping presentation of the two diseases on MRI suggests the importance of clinicians recognizing the features of both diseases. A comprehensive diagnostic strategy including genetic testing, neuroimaging, and detailed clinical evaluation is required.

## 1 Introduction

Wilson’s disease (WD) is an autosomal recessive disorder caused by mutations in the ATP7B gene, resulting in abnormal copper metabolism and accumulation in hepatocytes and extrahepatic organs, such as the brain and cornea. The global prevalence of Wilson’s disease was first estimated to be 1:200,000 by Scheinberg and Sternlieb in 1968, based on fragmentary data available at the time (1). In 1984, they updated their estimate to 1:3000 based on data from the United States Vital Statistics Reports 1968–1978, prevalence reports from clinical populations in East Germany, and epidemiologic analyses of close-knit families in Japan (2). In the literature to date, 1,275 ATP7B gene variants have been identified from 16,183 patients, with more than 80% of the variants originating from East Asia and Europe, while the remainder (less than 20%) are from North/South America, North Africa, and the Middle East-South Asia (3). Most patients are pure or compound heterozygotes for common mutations, with the most prevalent variant in East Asia being the missense R778L variant. It has been shown that the R778L variant is located in the exon 8 and transmembrane structural domain 4 (TM4) regions of the protein, affecting its localization and the transport of ATP7B (4). The most common mutation in patients from Central, Eastern and Northern Europe is the point mutation H1069Q (exon 14). Approximately 50–80% of WD patients in these countries carry at least one allele of this mutation, with allele frequencies ranging from 30 to 70% (5).

Clinical manifestations of WD in children can range from asymptomatic liver disease to cirrhosis or acute liver failure; neurologic and psychiatric symptoms are rare. In up to 60% of WD patients of all ages, the first symptom is liver disease, and older children and adolescents with WD may present with neurologic or psychiatric disorders. Basic diagnostic methods for WD in children include serum copper blue protein and 24-h urinary copper excretion. A symptom-based diagnostic scoring system, biochemical tests to assess copper metabolism, and molecular analysis of mutations in the ATP7B gene can be used to determine the final diagnosis of WD (6). Genetically, the diagnosis of WD requires either two disease-causing mutations or one disease-causing mutation in purity, but according to the guidelines of the American Association for the Study of Liver Diseases (AASLD), mutational analyses should be performed in individuals for whom the diagnosis is difficult to confirm by clinical and biochemical testing. The diagnosis of Wilson’s disease in the Leipzig score combines clinical symptoms, laboratory tests, and mutation analysis results.

Alexander disease (AxD) is a very rare neurodegenerative disorder caused by a dominant gain-of-function mutation in the glial fibrillary acidic protein (GFAP) gene located in chromosome 17q21, which is pathologically marked by the formation of cytoplasmic aggregates in astrocytes. The prevalence of Alexander disease is unknown; a nationwide epidemiologic survey in Japan showed a prevalence of AxD of approximately 1 case per 2.7 million people in Japan (7). The most common classification divides patients into three groups based on age of onset, infancy (0–2 years), adolescence (2–12 years), and adulthood (>12 years) (8). Among them, the neurological manifestations of juvenile Alexander disease usually include paralysis, medullary or pseudo-medullary signs, cerebellar ataxia, vegetative dysfunction, nystagmus, palatal myoclonus, and dementia, etc. MRI usually reveals signal abnormalities mainly focusing on 1, cerebral white matter signaling in frontal lobe regions; 2, signaling abnormalities of swelling or atrophy in the basal ganglia; 3, thalamus, periventricular rim, and brainstem lesions, and contrast enhancement; 4, “tadpole atrophy,” i.e., abnormal signaling or atrophy of the medulla, medulla oblongata, and/or cervical medulla (9, 10).

In addition to neurologic and MRI manifestations, a definitive diagnosis of AxD requires a combination of genetic analysis or pathologic diagnosis. Screening for GFAP mutations in key regions should facilitate the diagnosis of suspected cases of AxD (11). Recent insights suggest that blood GFAP has the potential to track even subtle structural CNS lesions in a variety of neurologic and systemic diseases (12). Recent studies using cellular and animal models have shown that the pathology of AxD involves functional abnormalities not only in intermediate filaments but also in astrocytes and neurons (13). A Japanese study categorized the clinical forms of AxD into the following three types based on neurological and MRI findings: cerebral (type 1), bulbospinal (type 2), and intermediate (type 3), with a suspected diagnosis when a patient fits into one of the types, and further genetic or pathologic testing to improve diagnostic sensitivity (7).

## 2 Case presentation

A 10-year-old girl from Shandong, China, came to our hospital with decreased speech, limb weakness, and difficulty walking in the past 2 months. She presented with mental behavioral abnormalities such as irritability and aggressive behavior in the last 20 days. The patient’s father complained on her behalf that the patient’s academic performance had declined significantly in the last 6 months. The patient had a negative Kayser-Fleischer ring, and we performed laboratory tests on her, which showed decreased copper oxidase (0.192 vital units/L), increased 24-h urinary copper excretion (280.67ug/24 h), and decreased copper cyanin (0.17 g/L). The patient had a prominent forehead, and her neurologic examination was characterized by difficulty in balance and walking, positive bilateral Babinski signs, hypertonic tendon reflexes, hypotonia, and grade IV muscle strength.

**Figure 1:**
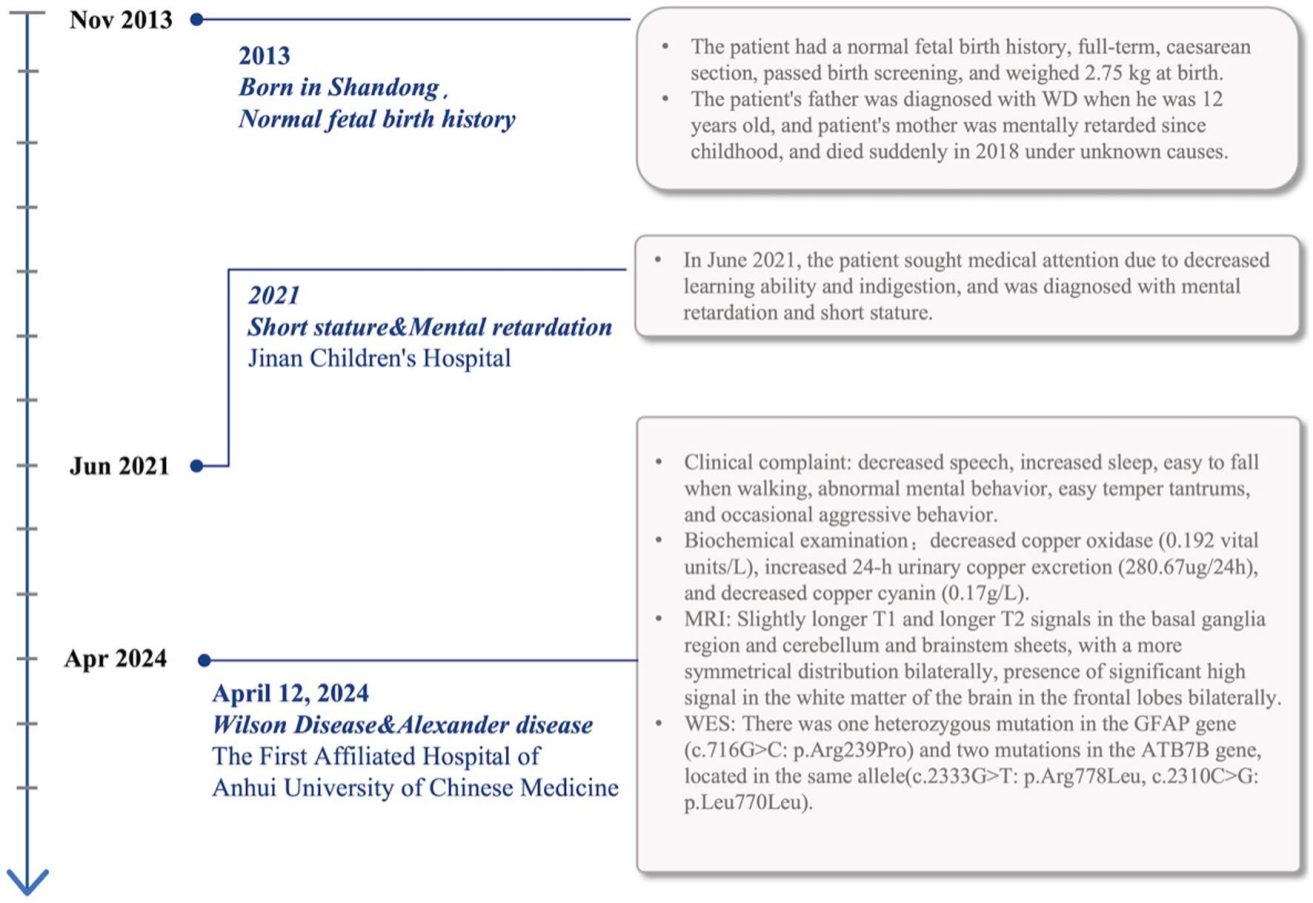
The patient’s course and associated diagnosis including signs, symptoms, and test results are noted on the timeline.

She started at the age of 6 years with developmental delay and mental retardation. She was diagnosed with cretinism and mental retardation in 2021 when she was seen at a local medical facility for declining academic performance. The patient’s mother, who had suffered from mental retardation since childhood, passed away when she was 4 years old, the cause of death was unknown, and her father had been diagnosed with Wilson’s disease (Figure 1). The combination of the patient’s neurologic symptoms and copper biochemistry findings, with a Leipzig score of 4, established the diagnosis of Wilson’s disease.

**Figure 2:**
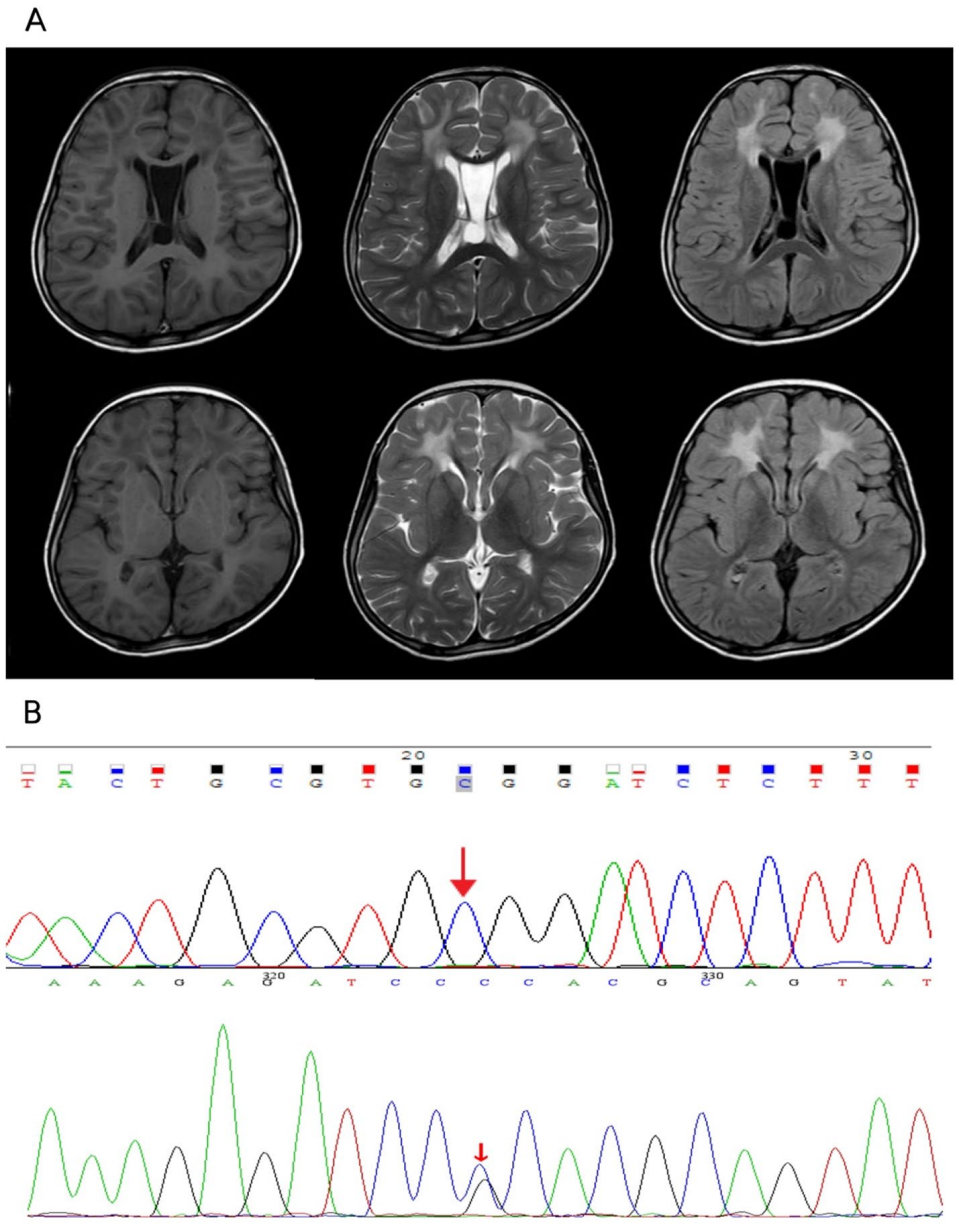
**(A)** Patchy slightly longer T1 and longer T2 signals were seen in the white matter of bilateral frontal lobe, bilateral basal ganglia area and cerebellum, brainstem, FLAIR showed higher signal, with a more symmetrical distribution bilaterally, corpus callosum was thin, part of the sulcus of bilateral cerebellar hemispheres was deep and wide, the interspace between the septum pellucidum was wider, the widest part was about 19.7mm, the fourth ventricle was wider, the occipital pools were larger, and no displacement of the midline structure was seen. A few T2WI high signal shadows were seen in the sinuses. **(B)** The patient had a monoallelic variant of the GFAP gene, while her father had no variation at this locus.

Slightly longer T1 and longer T2 signals in the basal ganglia region and cerebellum and brainstem patches were visualized by cranial magnetic resonance imaging tests, with a more symmetrical distribution bilaterally (Figure 2A). Notably, the patient also showed more symmetrical abnormal signals in the white matter of the frontal lobe bilaterally, which is unusual in patients with WD. Given that the bilateral frontal white matter lesions were not fully consistent with Wilson’s disease MRI, we performed whole-exome sequencing on the patient and her father to further clarify the diagnosis of this patient.

Whole-exome sequencing reported that the patient had a monoallelic variant of the GFAP gene (c.716G > C: p.Arg239Pro), whereby a guanine G on nucleotide 716 was changed to a cytosine C (c.716G > C), resulting in a change in amino acid 239 from arginine to proline (p.Arg239Pro). This variant is defined as suspected to be deleterious according to ACMG guidelines. Her father had no variation at this locus (Figure 2B). Double heterozygous variants in the patient’s ATP7B gene located on the same allele were also detected (Supplementary material), one of which was deleterious according to the ACMG guidelines (c.2333G > T: p.Arg778Leu), and the other variant was of unknown pathogenicity (c.2310C > G: p.Leu770Leu). In combination with the results of genetic testing and the diagnostic criteria for AxD cranial MRI developed by Van Der Knaap et al. in 2001 (9, 14), the patient was diagnosed with juvenile AxD combined with WD.

## 3 Discussion

From the beginning of the consultation with this patient, her most prominent clinical symptoms were a recent and sudden worsening of mood disorders and the presence of a significant decline in academic performance over the last 6 months, accompanied by weakness in the extremities and difficulty in walking. Mood disorders are the most common psychiatric manifestation of WD. Epidemiology suggests that between 20 and 60% of patients with WD develop depression, and the high prevalence of depression in WD may be related to the patient’s negative reaction to the diagnosis of WD as well as physical incapacitation due to neurological deficits (15). 8.3% of patients with WD suffer from personality changes and learning-related problems, respectively (16). In clinical work, psychobehavioral abnormalities do occur more frequently in WD patients with neurological symptoms, but it has not yet been conclusively established that WD has a psychiatry-specific clinical presentation. However, there is growing evidence that WD can manifest as psychiatric symptoms alone for many years, with hepatic and neurologic involvement becoming clinically apparent only after several years. Delays in diagnosis and treatment are common in these individuals (16).

This is because the clinical presentation of patients with WD varies widely and the characteristic clinical signs of WD, such as Parkinsonian symptoms, are often not apparent in childhood. In most cases, the diagnosis of WD in children needs to be determined by a combination of clinical features and laboratory parameters. Taking into account the patient’s family history, especially with the reference to his father’s diagnosis of WD, we screened the patient’s copper biochemistry and MRI in the presence of the typical manifestations of WD, and without performing a WES to test for the presence of the ATP7B gene variant in the patient, it can be confirmed that the patient has achieved a Leipzig score of 4, with 1 point for low copper cyanophorin, 2 points for high copper excretion, and 1 point for neurologic signs and symptoms, and that the diagnosis of WD has been adequately based.

According to a study characterizing AxD phenotypes and determining correlations with age of onset and gene mutations, type I AxD is characterized by early onset, seizures, macrocephaly, motor retardation, encephalopathy, growth retardation, paroxysmal deterioration, and typical MRI features. Type II is characterized by late onset, autonomic dysfunction, oculomotor abnormalities, medullary symptoms, and atypical MRI features (17). Our patient presented with clinical symptoms of both types of AxD, i.e., motor and cognitive delays, which are characteristic of early-onset AxD, and ataxia, which is characteristic of late-onset AxD, among other physical findings.

The significant high signal in the white matter of the bilateral frontal lobe of the brain that we observed in this patient is unusual in terms of the patient’s neuroimaging findings and has rarely been reported. Typically, white matter hyper-signal is seen in vascular dementia, and white matter hyper-signal has been associated with post-stroke cognitive dysfunction (18). Multiple sclerosis can exhibit white matter lesions, including frontal regions (19). However, both vascular dementia and multiple sclerosis are rare in young patients, making it easy to rule out this differential diagnosis.

This atypical neurological symptom of WD in children attracted some attention from us, based on the additional information provided by the patient’s father reviewing the child’s medical history, that the patient presented with neurological symptoms of motor and cognitive delays from the age of about 6 years, which were not emphasized at that time with any specific interventions. Considering the patient’s atypical WD changes in both neurologic symptoms and neuroimaging, we thought that we could not exclude the possibility of other neurologic disorders to the extent of overlapping symptoms. Given this complexity, we used WES in order to further confirm her genetic diagnosis, to troubleshoot the presence of other neurologic co-morbidities, or just one of the atypical psychiatric and imaging changes seen in pediatric patients with WD.

It is known in the WES report that our patients have double heterozygous variants in the ATP7B gene located on the same allele (c.2333G > T: p.Arg778Leu, c.2310C > G: p.Leu770Leu). Several studies on ATP7B gene mutations have been conducted in mainland China, all of which indicate that the Arg778Leu exon 8 mutation is a hotspot in the Chinese WD population. Phenotype–genotype correlation analysis showed that c.2333G > T (p.Arg778 Leu) was significantly associated with lower serum copper blue protein levels (*p* = 0.034) (20). However, mutations occurring in the TM4 structural domain of the ATP7B protein, i.e., c.2310C > G (p.Leu770Leu), are rare in WD patients, but there is still evidence in the literature, such as a 2011 statistic on the prevalence of mutations in the ATP7B gene in WD patients from Iran, where this *de novo* mutation had been reported (21). An analysis of the ATP7B gene in 65 WD patients in Hong Kong, China, revealed that p.R778L/p.L770L is a mutation specific to East Asia, dating back as far as 5500 years ago (22).

Based on the results of WES, the unusual imaging manifestations of this patient could then explain the overlapping changes of WD and AxD. AxD is a cerebral white matter dystrophy caused by mutations in the GFAP gene with characteristic symmetrical white matter abnormalities, predominantly in the frontal lobe (9), and we believe that the manifestations of white matter abnormalities on MRI in our patient are consistent with the cerebral white matter changes of Alexander’s disease. In addition, the bilateral slightly longer T1 and longer T2 signals in the basal ganglia region and the cerebellum and brainstem sheets with a more symmetrical distribution bilaterally that this patient had are also consistent with the characteristic neuroimaging changes of Wilson’s disease, which are usually found in the chiasmatic nuclei, thalamus, and brainstem (23). In contrast, involvement of the basal ganglia or thalamus can also be seen in adolescent-onset AxD (24). Frontal symmetric white matter hyperintensities are the hallmark imaging changes of AxD, usually with diffuse symmetric involvement of frontal white matter, with additional findings often seen in the basal ganglia, thalamus, and periventricular regions. The symmetry of the frontal lobe lesions observed in our patient fits well with the known magnetic resonance imaging features of AxD, which is precisely what prompted us to consider a comorbid diagnosis at that time. Although white matter abnormalities are rare in WD, isolated cases have been reported. Zhong et al. reported a 5.9% incidence of WD MRI abnormalities occurring in the cerebral cortex and white matter (25). A few reports suggest that WD white matter lesions generally favor the frontal lobes, especially precentral regions (26). Magnetic resonance imaging (MRI) shows WD cerebral white matter lesions that are usually asymmetric (27), which is dissimilar to the white matter abnormality in our patient, but there have been case reports of symmetric white matter hyper-signal involving the frontal lobe and parieto-occipital regions (28). One study reported abnormal variants in patients with AxD that included asymmetric frontal white matter abnormalities, basal ganglia abnormalities, and multiple tumor-like brainstem lesions (10). There also exist reports of AxD patients with cranial MRI without cerebral hemispheric white matter and basal ganglia abnormalities, while their lesions symmetrically involve the brainstem, cerebellum, and spinal cord (29).

The diagnostic process is further complicated by imaging findings in the mid-basal ganglia, cerebellum, and brainstem regions. The more symmetrical high signal posted in these regions is generally easy to recognize as a typical neuroimaging manifestation of WD based on the patient’s family history and copper biochemical findings, reflecting copper deposition and resulting neuronal damage. However, AxD may also involve the basal ganglia, brainstem, and cerebellum, and studies have reported symmetric or tumor-like lesions in these regions in patients with AxD. In addition, MRI showed a thin corpus callosum in this patient, and according to the literature, corpus callosum abnormalities are not uncommon in WD, with an incidence of 23.4% (30). A study from Anhui, China, attempted to correlate neuroimaging observations of cerebral white matter abnormalities in WD with clinical symptoms, and its results showed that damage to the corpus callosum and corticospinal tract microstructures may be involved in the pathophysiologic process of neurological symptoms in WD, such as may be associated with clinical symptoms such as gait and balance deficits, involuntary movements, dysphagia, and autonomic dysfunction (31).

Symmetrical white matter lesions, although characteristic of AxD, may also be present in WD, albeit rarely, as confirmed by published case reports. Similarly, symmetrical basal ganglia, cerebellar and brainstem abnormalities, although typical of WD, are not unique to WD and may represent AxD pathology. Patients with WD who have psychiatric symptoms usually have a history of misdiagnosis with an initial diagnosis of schizophrenia, schizoaffective, or delusional disorder. Psychobehavioral abnormalities and cognitive developmental delays are also usually common clinical manifestations of adolescent-type AxD. Psychotic symptoms presenting as the first manifestation of WD may present diagnostic and treatment challenges (32). Because approximately 3% of first psychiatric symptoms may be due to “organic” causes (e.g., copper toxicity in WD), some diagnostic guidelines recommend screening for WD in patients whose initial symptoms are psychiatric (33). The coexistence of these overlapping clinical symptoms and imaging manifestations emphasizes the importance of a multidisciplinary approach to diagnosis in such cases.

WD is one of the few metabolic disorders that can be successfully treated pharmacologically with early diagnosis (34). Early diagnosis can be achieved through family screening of first-degree relatives of patients with WD and differential diagnosis of other liver and movement disorders, which can lead to proper treatment of the disease (35). It should be emphasized that one of the most important determinants of poor treatment outcomes is delayed diagnosis. Patients with Wilson’s disease with predominantly neuropsychiatric symptoms tend to present other clinical symptoms later, resulting in a longer delay from symptom onset to definitive diagnosis and a poorer prognosis than patients with hepatic symptoms. It can be argued that in both WD and AxD, clinical symptoms are the starting point for diagnosis, especially in such complex co-morbidities. The patient’s neurologic manifestations such as mood disorders, cognitive delays and ataxia provide important clues for the initial evaluation. For atypical or overlapping symptoms (e.g., abnormal bilateral frontal white matter signal in this patient), one should be wary that a single diagnosis is insufficient to explain the condition. In pediatric patients, especially cases with concomitant neuropsychiatric symptoms, it is recommended to optimize biochemical screening criteria to enhance sensitivity in conjunction with family history. MRI showed rare white matter and basal ganglia signal abnormalities in this case, providing an important basis for a dual diagnosis. This suggests that in our routine evaluation for atypical imaging manifestations, we should pay attention to the imaging overlap between WD and other cerebral white matter lesions (e.g., AxD complicating this patient) to avoid missed diagnosis. The application of WES in this case not only revealed the ATP7B and GFAP gene mutations, but also verified a comprehensive diagnosis of imaging and biochemical findings. In the absence of characteristic clinical manifestations, or when the case is complex (e.g., multi-system symptoms or overlapping MRI manifestations), WES can be used as a complementary tool to clarify the diagnosis and assist in the development of an individualized treatment plan.

In summary, from the experience of diagnosis and treatment of this patient, we recommend a multistep diagnostic approach that integrates clinical findings, biochemical testing, imaging analysis and genetic analysis. Considering the patient’s mother’s history of mental retardation since childhood, because of her early death and the lack of information about her visits, it has not yet been possible to exclude the possibility that her mother suffered from Alexander’s disease, which limits our ability to further analyze the patient’s genetic family pedigree. In future practice, a detailed history of the patient’s illness and family genetic history should be refined, the patient’s neurological and psychiatric status should be monitored and evaluated, and WES monitoring should be considered to enhance the credibility of the WD diagnosis when there are abnormalities in copper biochemistry tests, psychiatric anomalies, and atypical changes in neuroimaging and to exclude the possible remaining disorders, so as to comprehensively assess and analyze the patient’s prognosis and management options. This strategy not only highlights the central role of imaging and genetic testing in making a definitive diagnosis, but also emphasizes a multidisciplinary diagnostic perspective when multiple neurological disorders coexist. Especially in cases with overlapping or atypical characteristic presentations, WES analysis can serve as an important link for final confirmation, significantly improving diagnostic accuracy. This step-by-step approach is of great significance for the accurate diagnosis of complex cases in the field of neurology and provides a reliable basis for the optimization of subsequent treatment plans.

## Data Availability

The datasets presented in this study can be found in online repositories. The names of the repository/repositories and accession number(s) can be found in the article/Supplementary material.
