# Supplementary material for: Case report: Co-occurrence of Wilson’s and Alexander’s diseases revealed by genetic analysis
Source: Front Neurol. 2025 Mar 12;16:1514044. doi: 10.3389/fneur.2025.1514044 (PMC11938389; doi:10.3389/fneur.2025.1514044)

Supplementary Material

**Supplementary Table 1.** Identification of the patient’s genetic mutations and variants.

| **Gene name** | **GenBank ID: Exon: Nucleotide change: Amino Acid change** | **State** | **Variation type** | **Inheritance pattern** | **ACMG grade** | **Disease/phenotype** |
| --- | --- | --- | --- | --- | --- | --- |
| *ATP7B* | NM_000053:exon8:c.G2333T:p.R778L | Hetorozygous | Missense | AR | Pathogenic | Wilson disease |
| *ATP7B* | NM_000053:exon8:c.C2310G:p.L770L | Hetorozygous | Missense | AR | Uncertain | Wilson disease |
| *GFAP* | NM_002055.5:exon4:c.G716C:p.Arg239Pro | Hetorozygous | Missense | AD | Likely Pathogenic | Alexander Disease |
| *SLC22A5* | NM_003060.4:exon8:c.C1400G:p.Ser467Cys | Hetorozygous | Missense | AR | Likely Pathogenic | Systemic primary carnitine deficiency |
| *MYH2* | NM_017534.6;intron5:c.T505+2C:p.? | Hetorozygous | Missense | AD,AR | Likely Pathogenic | Congenital myopathy with ophthalmoplegia |

AR: autosomal recessive; AD: autosomal dominant.

**Supplementary Figure**

**Figure 1.** The patient's course and associated diagnosis including signs, symptoms, and test results are noted on the timeline.


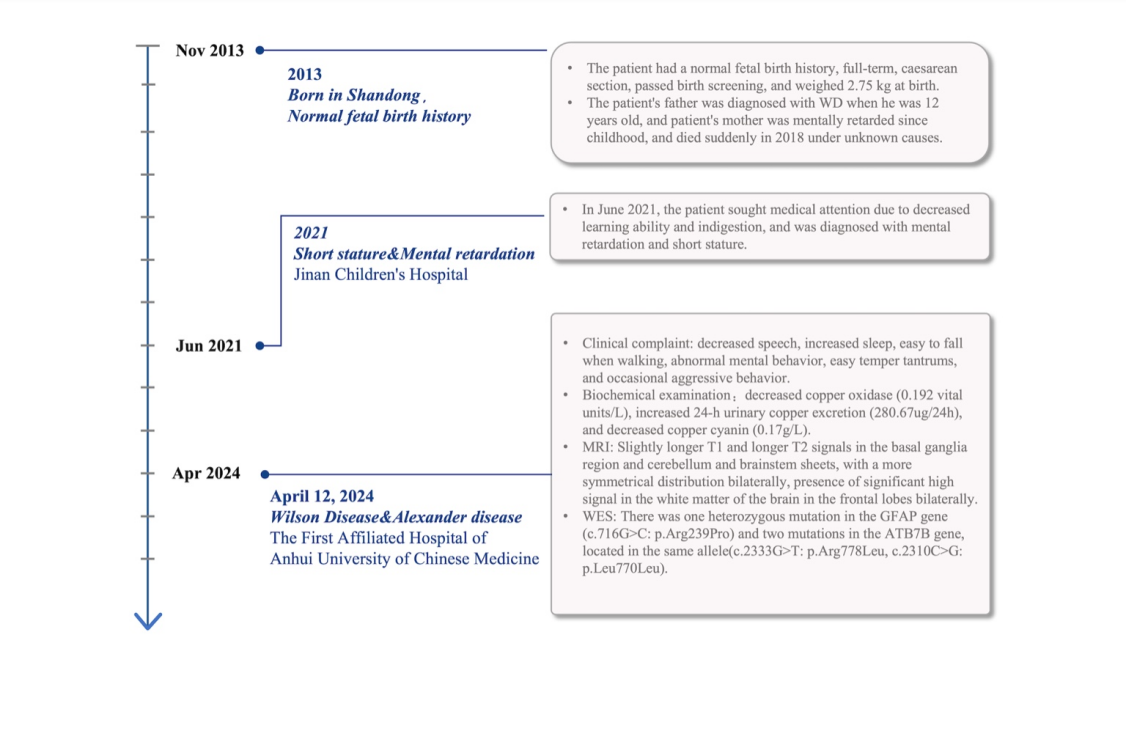


**Figure 2A.** Patchy slightly longer T1 and longer T2 signals were seen in the white matter of bilateral frontal lobe, bilateral basal ganglia area and cerebellum, brainstem, FLAIR showed higher signal, with a more symmetrical distribution bilaterally, corpus callosum was thin, part of the sulcus of bilateral cerebellar hemispheres was deep and wide, the interspace between the septum pellucidum was wider, the widest part was about 19.7mm, the fourth ventricle was wider, the occipital pools were larger, and no displacement of the midline structure was seen. A few T2WI high signal shadows were seen in the sinuses.

**Figure 2B.** Thepatient had a monoallelic variant of the GFAP gene, while her father had no variation at this locus.


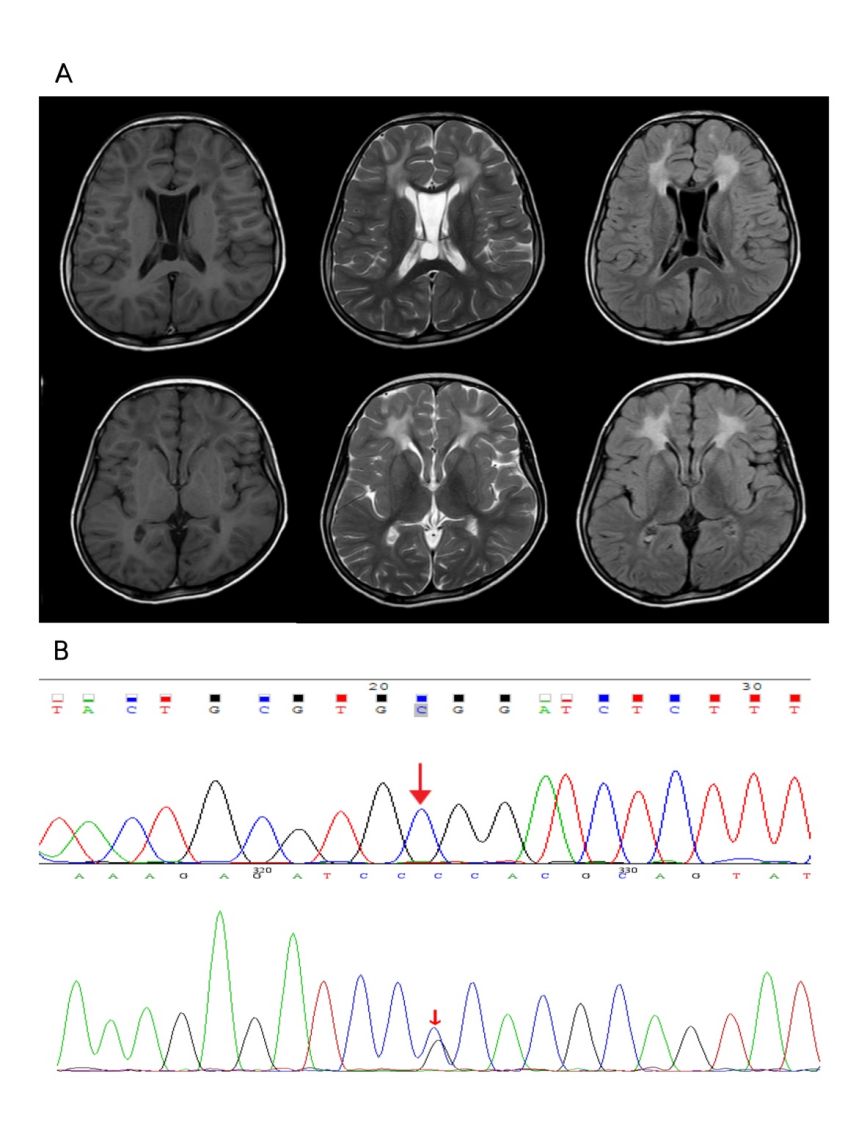

Supplement: Supplementary file 1 [file Table_1.docx]
